# Jellyfish-Associated Microbiome in the Marine Environment: Exploring Its Biotechnological Potential

**DOI:** 10.3390/md17020094

**Published:** 2019-02-01

**Authors:** Tinkara Tinta, Tjaša Kogovšek, Katja Klun, Alenka Malej, Gerhard J. Herndl, Valentina Turk

**Affiliations:** 1 Department of Limnology and Bio-Oceanography, University of Vienna, Althanstrasse 14, A-1090 Vienna, Austria; gerhard.herndl@univie.ac.at; 2 Marine Biology Station Piran, National Institute of Biology, Fornače 41, 6330 Piran, Slovenia; tjasa.kogovsek@nib.si (T.K.); katja.klun@nib.si (K.K.); alenka.malej@nib.si (A.M.); valentina.turk@nib.si (V.T.); 3 NIOZ, Department of Marine Microbiology and Biogeochemistry, Royal Netherlands Institute for Sea Research, Utrecht University, 1790 AB Den Burg, The Netherlands

**Keywords:** Cnidaria, Ctenophora, biodiversity, bioactive compounds, microbial communities, blue biotechnology

## Abstract

Despite accumulating evidence of the importance of the jellyfish-associated microbiome to jellyfish, its potential relevance to blue biotechnology has only recently been recognized. In this review, we emphasize the biotechnological potential of host–microorganism systems and focus on gelatinous zooplankton as a host for the microbiome with biotechnological potential. The basic characteristics of jellyfish-associated microbial communities, the mechanisms underlying the jellyfish-microbe relationship, and the role/function of the jellyfish-associated microbiome and its biotechnological potential are reviewed. It appears that the jellyfish-associated microbiome is discrete from the microbial community in the ambient seawater, exhibiting a certain degree of specialization with some preferences for specific jellyfish taxa and for specific jellyfish populations, life stages, and body parts. In addition, different sampling approaches and methodologies to study the phylogenetic diversity of the jellyfish-associated microbiome are described and discussed. Finally, some general conclusions are drawn from the existing literature and future research directions are highlighted on the jellyfish-associated microbiome.

## 1. Introduction

### 1.1. Biotechnological Potential of Host–Microorganism Systems in the Ocean

Our fascination with the hidden treasures of the ocean is evident since Jules Verne’s classic masterpiece Twenty Thousand Leagues Under the Seas, but only recently have we started to understand and unravel the incredible biotechnological potential of the ocean, in particular the potential locked in the vast diversity of marine microorganisms. Oceans represent the largest biosphere on the planet, and their smallest but most abundant, productive, and diverse residents, marine microorganisms, inhabit every marine habitat; with their diverse metabolic pathways, they play a central role in biogeochemical cycles in the oceans [1,2,3]. Consequently, marine microorganisms are potentially also a source of biotechnologically important enzymes, other compounds, and molecules [4,5]. For example, polymer-degrading enzymes and robust enzymes isolated from extremophilic microorganisms are being successfully applied in several branches of industry, from laundry detergents and food processing to sophisticated molecular biology reagents. Biosurfactants and (extracellular) polymeric substances from marine bacteria are also finding increasing applications in bioremediation, industrial processes, manufacturing, and food processing and are used as underwater surface coatings, bio-adhesives, drag-reducing coatings on ship hulls, dyes, sunscreens, biodegradable plastics, etc. (reviewed in Reference [6]). In addition, a large set of bioactive compounds from marine microbes has been tested for their biomedical potential, such as antibacterial, antifungal and antiviral agents, anticancer and anti-inflammatory drugs, drug delivery agents, and others (reviewed in References [7,8,9,10]). In addition, it seems that the more peculiar, rich, or extreme their habitat is, the more biotechnologically interesting molecules microorganisms produce.

In the ocean, where microorganisms are constantly facing changing environmental conditions on a microscale level, one of the adaptation/survival strategies of some microbes is to establish long-term relationships with other organisms. At the same time, these host-microorganism systems are production hotspots of chemical compounds and/or secondary metabolites that can serve as the system’s own defense mechanism against predators, colonization, and/or disease [11,12,13,14]. It is becoming evident that many of the bioactive compounds isolated from these systems are actually a result of the microorganisms’ rather than the host’s biosynthesis/metabolism or the interaction of both. Therefore, explorations of the taxonomic and metabolic diversity of the host-associated microbiome and investigations into mechanisms underlying these associations provide answers to questions regarding the evolution and ecology of these systems. This research is also generating datasets that can be screened for novel microbial strains, genes, secondary metabolites, byproducts, and other compounds of microbial (and host) origin that could be exploited by the fast-growing blue biotechnology sector, in a process known as bioprospecting [1].

### 1.2. Gelatinous Zooplankton as Host for Specific Microbiome

Marine invertebrates have been extensively studied as hosts of microorganisms producing compounds with biotechnological potential (e.g., [11,12,14,15,16]). Recently, among the marine invertebrates, Porifera (sponges), Annelida (within which are Polychaeta, marine worms), and Cnidaria (within which are corals, mostly octocorals) have been investigated, focusing on their associated microbiome and potential biotechnological applications [17]. The biotechnological potential of the cnidarians and their interactions with their microbiome was recently reviewed ([18] this issue), with the main focus on the coral holobiont. In addition, cnidarian–microbe interactions were investigated in detail in *Hydra* as a model host and its holobiont [19]. Medusozoans, characterized by the presence of a pelagic phase in their life cycle, are less well studied. To the best of our knowledge, medusozoans have never been comprehensively reviewed in terms of their interactions with their microbiome or as hosts of a microbiome with biotechnological potential.

In this review, we use the term “jellyfish” to describe gelatinous marine plankton belonging to the cnidarian subphylum Medusozoa (Scyphozoa, Cubozoa, and Hydrozoa) and phylum Ctenophora. Their convergent features are transparency and fragility, their body surface is coated with mucus, and they lack a hard skeleton. In addition, the proteinaceous body has a high water content (>95%) accompanied by a low content of organic matter on a wet mass basis. Ctenophora spend all their life in the pelagic environment, while the life cycle of a large majority of Medusozoa is characterized by a shift of planktonic medusa and a benthic polyp phase. Therefore, the Medusozoa-associated microbiome experiences changes in terms of morphology and biochemical structure of their hosts and a complete shift of the hosts’ lifestyle from benthic/attached to pelagic/swimming/free-living. Another important fact to consider is that as the medusa stage drifts with ocean currents over long distances, its associated microbiome could be subjected to changing environmental conditions as could represent pressure on the new environment (e.g., jellyfish as vectors of allochthonous microbes’ transmission in the marine environment). Taking into account their unique simple anatomy, evolutionary age, alteration between different life stages, wide distribution, and important role in diverse marine ecosystems worldwide, Medusozoa and Ctenophora could potentially harbor taxonomically and metabolically diverse microorganisms with great biotechnological potential. This seems even more important (and justified) in light of the recently reported increase of jellyfish blooms in several marine environments [20], with potentially serious ecological and socio-economic consequences. Due to high reproductive output and fast growth, jellyfish form blooms when conditions are favorable, reaching high biomass within a short period of time ([21] and references therein), and represent a major source of organic matter for the marine ecosystem. It was shown that jellyfish blooms influence the diversity of marine food webs and may affect biogeochemical cycles in the ocean [21,22,23,24]. Especially at the end of their lifespan, jellyfish debris represents hotspots for growth and development of specific, even pathogenic, microbial phylotypes, potentially affecting human health and well-being [25,26,27,28,29].

The aim of this paper is to critically review the existing literature on the microbiome associated with jellyfish in terms of: (i) screening which jellyfish taxa have already been investigated for their associated microbiome and how many remain to be explored; (ii) different methodological approaches applied to study the jellyfish-associated microbiome; (iii) understanding the characteristics of the jellyfish-associated microbiome in terms of (a) degree of microbiome specialization (e.g., generalist versus specialist), (b) preference of the microbiome for specific jellyfish taxa, and (c) specificity of the microbiome at the jellyfish population, (d) life stage, and (e) body part level; (iv) gaining insights into the composition and function of the jellyfish-associated microbiome; and (v) determining the biotechnological potential of the jellyfish-associated microbiome.

## 2. Jellyfish-Associated Microbiome

Thus far, only a limited number of studies have focused on the microbiome associated with jellyfish during their life span (Table 1). Early reports on microbes associated with jellyfish were simple observations, where jellyfish was a primary object of research [30,31,32]. Later, studies specifically focused on selected pathogenic bacteria associated with jellyfish, e.g., investigating them as vectors of fish pathogens [33,34]. Already these studies addressed questions on the ecology, composition, and role of microbial communities associated with jellyfish, the mechanisms underlying these interactions, and the nature of the relationships between jellyfish and their associated microbiome. Based on this, researchers have started to focus on the microbial counterpart of jellyfish–microbe associations, aiming at assessing the diversity of microbial communities associated with different jellyfish species from various ecosystems and with their different life stages and body compartments.

### 2.1. Microbiome Associated with Specific Jellyfish Taxa

Different taxonomic groups of jellyfish were studied for their associated microbiome, but maybe even more important, the vast diversity of jellyfish as hosts remain to be explored.

#### 2.1.1. Medusozoa

Within Medusozoa, most studies were performed on Scyphozoa, some on Hydroza and, to our knowledge, only one study on Cubozoa was published so far (Table 1).

#### 2.1.2. Scyphozoa

Inside the Scyphozoa class, also known as the “true jellyfish”, only members from Rhizostomeae and Semaeostomeae orders have been investigated for their associated microbes so far.

#### 2.1.3. Semaeostomeae

Within Semaeostomeae members of Ulmaridae (*Aurelia aurita*, or moon jellyfish, [35,36,37]), Cyaneidae (*Cyanea capillata*, known as lion’s mane jellyfish, and *Cyanea lamarckii*, or blue fire jellyfish [38,39]) and Pelagiidae (*Pelagia noctiluca*, known as mauve stinger [33], *Chrysaora plocamia*, a South American sea nettle [40], and *Chrysaora hysoscella*, compass jellyfish [39]) were studied for their associated microbial communities.

#### 2.1.4. Ulmaridae

One of the most studied jellyfish is *Aurelia aurita*, also known as moon jellyfish. To our knowledge, it is the only jellyfish species from this family that has been investigated for its associated microbiome, and at the same time the most comprehensively investigated of all jellyfish species. Nevertheless, we have to point out the unclear taxonomy of genus *Aurelia*. Mayer [46] and Kramp [47] described 12 and 6 *Aurelia* species, respectively, based on morphological characteristics of the medusa; later only *A. aurita* and *A. labiata* were recognized as distinct species [48]. Despite more than 100 years of *Aurelia* research, the taxonomy of this genus is still unclear [49], and recent molecular analysis indicated that *A. aurita* is represented by several cryptic species [50,51]. The World Register of Marine Species (WoRMS) currently (as of 17 December 2018) recognizes nine *Aurelia* species. It is therefore not always clear which of these species are investigated for their associated microbiome, but for this review we will retain the species name given by the authors of the respective articles.

The first and most detailed study of the microbiome of *A. aurita* was performed by Weiland-Bräuer et al. [35]. These authors investigated the microbiome associated with different life stages of *A. aurita* (polyp, strobila, ephyrae, juvenile, and adult medusae), investigated different compartments of the adult medusae (mucus versus gastric cavity), and compared the microbiome of the polyp stage of specimens from different geographic locations. In this study, fluorescence in situ hybridization (FISH) was used to determine the distribution of specific groups of bacteria on the polyps. The taxonomic composition of the microbiome of *A. aurita* was assessed using the next generation sequencing technique 454 pyrosequencing of the V1–V2 hypervariable region of the 16S rRNA gene.

While several interesting findings were reported in the study of Weiland-Brauer et al. [35], here we highlight only those most important for our review. Different stages of the strobilation event, strobila and ephyra, and juvenile medusa harbored a similar microbiome, which significantly differed from the microbiome of the non-strobilating perennial polyp. At the same time, the richness of the bacterial community was approximately the same among all examined life stages. The microbiome analysis revealed that *Gammaproteobacteria*, *Alphaproteobacteria*, *Bacteroidetes*, and *Actinobacteria* dominate all life stages, with different relative contributions of each bacterial group depending on the stage. Based on 16S rRNA gene amplicon sequencing and FISH coupled with confocal laser scanning microscopy, the authors reported that the entire epithelial surface of the polyp stage of *A. aurita* was covered by bacteria. They proposed that colonization of the polyp occurs on the mucus coating its epithelial surface, mainly by *Gammaproteobacteria* (mainly *Crenothrix*), while bacteria detected inside and between cells of the polyp tissue were most likely a novel *Mycoplasma* strain (class *Molicutes*), suggested to be potential endosymbionts of *A. aurita* polyps. However, sequencing approaches failed to detect this strain in polyps. Other dominant colonizers of polyps were *Bacteroidetes* (in particular *Lacinutrix*, a member of the *Flavobacteriaceae* family) and *Alphaproteobacteria* (dominated by *Phaeobacter*, a member of the *Rhodobacteriaceae* family). The next developmental phase of *A. aurita*, the strobilating polyp, was associated with *Gammaproteobacteria* (*Crenotrichaceae* and *Vibrionaceae*) and *Actinobacteria* (*Nocardiaceae*). Newly released ephyrae were dominated by *Gammaproteobacteria* (*Crenotrichaceae* and *Pseudoalteromonadaceae*), *Alphaproteobacteria* (*Rhodobacteraceae*) and *Actinobacteria* (*Microbacteriaceae*). Juvenile medusae were dominated by *Alphaproteobacteria* (*Rhodobacteraceae*), *Flavobacteriaceae*, and *Gammaproteobacteria* (*Vibrionaceae*). The differences in bacterial community composition between gastric cavity and umbrella mucus were also determined, revealing that both compartments were dominated by unclassified *Mycoplasma*. A small number of bacterial sequences from the gastric cavity and mucus were affiliated with *Rhodobacteraceae* (*Alphaproteobacteria*). Bacterial diversity differed between the gastric cavity and mucus, with mucus exhibiting greater variability in richness. The drawback of this study is the relatively low number of samples analyzed. One should be careful in extrapolating these results to the entire *A. aurita* taxonomic group (or even beyond), in particularly, since this study also shows the effect of the host’s natural environment on the composition of jellyfish-associated microbiome.

The other two available studies of the microbiome of *A. aurita*, from the North Atlantic Ocean [36] and the Northern Adriatic [37], investigated only the adult medusa stage. Both Daley et al. [36] and Kos Kramar et al. [37] constructed and sequenced bacterial 16S rRNA gene clone libraries, which means that compared to the next-generation sequencing approach used by Weiland-Bräuer et al. [35], the number of sequences obtained and analyzed was lower, hence the entire diversity of bacteria was most likely not captured, particularly that of the rare community members. At the same time, the bacterial 16S rRNA gene clone libraries resulted in long and good-quality sequences, allowing the classification to lower taxonomic levels. However, even these two studies using clone libraries cannot be compared entirely, as they used different bacterial primers to amplify different regions of 16S rRNA. Besides, Kos Kramar et al. [37] also applied a culturing approach to investigate the cultivatable part of the jellyfish-associated microbial community.

Daley et al. [36] analyzed seven adult specimens, all collected during two sampling days in spring of the same year. In contrast, Kos Kramar et al. [37] collected 20 adult individuals during two sampling events in the same year, one in spring and one in early summer, investigating changes of the *A. aurita*-associated microbiome in relation to the age of the jellyfish population. Each jellyfish sample pool was then split in half for a culture-based and culture-independent approach to analyze the composition of the *A. aurita*-associated microbial community [37]. For both studies, it is difficult to tell whether the analyzed sample pool is truly representative of the studied jellyfish population, but both sampling designs did not account for interannual variability or spatial patchiness.

The conclusions drawn from these two studies differ. While Daley et al. [36] showed that *Aurelia* is associated with a consortium of bacteria composed of *Mycoplasmatales* (*Tenericutes*, *Mollicutes*) and many unclassified bacteria, several of them distantly related to *Mycoplasma*, in line with the results of Weiland-Brauer et al. [35], Kos Kramar et al. [37] did not detected any bacteria affiliated with this taxonomic group. Also, while in the North Atlantic *A. aurita* harbored only few *Gammaproteobacteria* (including *Psychrobacter* spp.), *A. aurita* collected in the Northern Adriatic were dominated by the gammaproteobacterial families *Vibrionaceae*, *Pseudoalteromonadaceae*, *Xanthomonadaceae*, and *Pseudomonadaceae*. Within the *Alphaproteobacteria*, different families were associated with Aurelia from the two studied systems: in the North Atlantic *Rickettsiales*, and in the Northern Adriatic *Rhodobacteraceae* (mostly *Phaeobacter* and *Ruegeria*). In the Northern Adriatic, *Betaproteobacteria* and *Actinobacteria* were found associated with *A. aurita*, but were not detected in *A. aurita* from the North Atlantic while *Cyanobacteria* were associated with *A. aurita* in both the Northern Adriatic and the North Atlantic.

The body part-specificity of the *A. aurita* microbiome was also studied in the Northern Adriatic [37]. *Betaproteobacteria* dominated in the gastral cavity, while *Alphaproteobacteria* and *Gammaproteobacteria* dominated in the “outer” body parts, mostly ex-umbrella mucus, in accordance with Weiland-Brauer et al. [35]. Bacterial strains associated with polycyclic aromatic hydrocarbons (PAHs) and plastic degradation, such as *Stenotrophomonas, Pseudomonas, Burkholderia, Achromobacter*, and *Cupriavidus* [52,53,54,55,56], were present in *A. aurita*’s gastric cavity, indicating an adaptation to anthropogenic pollution. Furthermore, the bacterial community associated with *A. aurita* changed at the transition from the peak to the senescent phase of the jellyfish bloom, and was characterized by an increase of *Gammaproteobacteria*, especially *Vibrionaceae* and *Alteromonadaceae* [37]. Those authors speculated on the different roles that *Vibrionaceae* might play during different stages of jellyfish, from possible commensal opportunistic visitors at the peak of jellyfish bloom to consumers of moribund jellyfish biomass at the end of the jellyfish lifespan. This is in accordance with studies of the degradation of jellyfish biomass by ambient microbial communities, where *Vibrionaceae* were found to dominate the jellyfish biomass-degrading assemblages [25,26,27,28,29]. Furthermore, the authors suggested that *Vibrionaceae* could be exploiting the nutrient-rich niche provided by *A. aurita*. Under proper conditions, such as a disturbed defense mechanism of the jellyfish and elevated water temperatures at the end of jellyfish bloom, upregulating the determinants of *Vibrio*’s virulence, such as motility, resistance to antimicrobial compounds, hemolysis, and cytotoxicity [57], they could outcompete other bacteria and become highly dominant in *A. aurita*.

#### 2.1.5. Cyaneidae

The first insights into the microbiome of Scyphozoa were published by Schuett and Doepke [38] focusing on the pathogenic potential of endobiotic bacteria associated with the tentacles of *Cyanea capillata* and *Cyanea lamarckii*. At the same time, they examined one Hydrozoa (discussed below in the Hydrozoa section) and one sea anemone (not discussed in this review) using culturing and culture-independent approaches.

The data from culturing and culture-independent approaches showed that most of the bacteria associated with both Cyaneidae species were *Gammaproteobacteria*, exhibiting >97% similarity to the *Vibrio* group and closely affiliated with *V. splendidus/V. tasmaniensis/V. lentus/V. kanaloa*. Bacteria affiliated with *Pseudoalteromonas tetradonis/P. elyacovii/P. haloplanctis, Photobacterium profundum, Shewanella violacea, Shewanella sairae/S. marinintestina, Sulfitobacter pontiacus*, and *Arcobacter butzleri* were found to be exclusively associated with *C. capillata* tentacles. In contrast, *Moritella viscosa* and *Mesorhizobium tianshanense* were only found associated with *C. lamarckii*. The authors unfortunately did not provide information on the relative abundance of these specific bacterial groups. As no remarkable similarities between communities associated with specific jellyfish were detected, the authors suggested that the associated microbiome could be host-specific to some extent. However, as there was no attempt to analyze the bacterial community of the ambient seawater, we believe that the information available is not sufficient to support this conclusion.

The culture-independent approach should have described the diversity of the bacterial community associated with *C. lamarckii* and *C. capillata* to a great extent. However, the authors reported that the number of denaturing gradient gel electrophoresis (DGGE) bands, representing the richness of the bacterial community, was lower than the number of bands obtained using a preculturing step (see Reference [38] for details on study design). One possible explanation is that the abundance of bacteria on jellyfish tentacles is low and the preculturing step increased their biomass and consequently the DNA extraction yield. The authors reported many high-quality sequences exhibiting low similarity to any of the sequences available in the National Center for Biotechnology Information (NCBI) database at that time (<97% similarity). Therefore, as they were not able to show an affiliation of the phylogeny of these bacteria using the tools available at the time, they suggested that these bacteria should be considered as potentially new species. Their preculturing step, however, was probably selective toward a cultivatable jellyfish-associated microbiota, which might represent only a minor fraction of the total microbial community. In addition, as information on the number of jellyfish individuals, the amount of analyzed tentacles, and the number of replicates is not provided, it is difficult to evaluate how representative these results are. From the description of sampling, the analyzed individuals were all collected at one location, during one season, and over three consecutive years. It is not clear, however, which jellyfish were collected when and whether specific species were collected over different years. Thus, the interannual variability could not be assessed.

The second study on *C. lamarckii* was published by Hao et al. [39]. This study provides valuable insights into the microbial community associated with different *Cyanea* body parts, revealing significant differences between bacterial communities associated with the umbrella and other parts, i.e., gonads, mouth, arm, and tentacles. The bacterial diversity was higher on the umbrella than on the mouth, arms, tentacles, and gonads. Altogether these results suggest a certain degree of body part specificity in the *Cyanea*-associated microbiome. The life stage specificity of the associated bacterial community was also investigated. Significant differences between bacterial communities associated with different jellyfish life stages were apparent, with the community’s richness increasing from larval stage to adult medusa. Furthermore, it was found that the type of food fed to the polyps affected the structure of the associated bacterial community.

The number of specimens analyzed in this study was high (*n* = 44) and samples were collected frequently within a short time interval (twice per week), accounting for the variability within the population, but only during one season. At the same time, spatial, seasonal, or interannual variability was not assessed. This is important, especially in light of reports on interannual fluctuations and large spatial variability for Scyphomedusae in the study region [58]. Furthermore, jellyfish analyzed in this study were collected using 500 µm mesh trawls, introducing potential cross-contamination with microbes associated with other biological material accumulated in the cod end of the net. The data on microbial community associated with *C. lamarckii* tentacles from the two available studies cannot be compared, unfortunately, since the taxonomic composition of the microbial community was not analyzed by Hao et al. [39] and the data obtained by DGGE [38] and automated ribosomal intergenic spacer analysis (ARISA) [39] are not comparable due to the different resolutions of these two fingerprinting techniques.

#### 2.1.6. Pelagiidae

Within the Pelagiidae family, jellyfish from genus *Chrysaora* have been most extensively studied, while only one study on the microbiota of *Pelagia noctulica* was published, focusing on a single specific bacterium, the fish pathogen *Tenacibaculum maritimum* (see Section 3.6 and Reference [33]). We did not find any studies on jellyfish from the genera *Mawia* and *Sanderia*.

The microbiome of *Chrysaora* was analyzed by Lee et al. [40], focusing on the benthic life stages of *Chrysaora plocamia*. In particular, forms involved in propagation through cyst formation, i.e., the mother polyp, its dormant cysts (podocysts), and polyps recently excysted from podocysts, were studied. The microbial community was analyzed using Illumina MiSeq sequencing of the V1–V2 region of the 16S rRNA gene. *Chrysaora plocamia* was collected along the Chilean–Peruvian Humboldt Current System (along the coast of Northern Chile) at different sampling times, accounting for temporal/seasonal variability. Polyps were grown in the lab from planulae subtracted from oral arms of collected jellyfish and produced podocysts, often followed by strobilation. No comparison with the bacterial community of the ambient seawater community was made, however, which would have allowed us to understand the degree of specialization of jellyfish-associated microbiome.

At a broader taxonomic level, the microbiomes of all life stages of *C. plocamia* were dominated by *Gammaproteobacteria* (mainly *Alteromonadales, Legionellales*, and *Methylococcales*), followed by *Alphaproteobacteria* (mostly *Rhizobiales*), *Bacteroidetes* (*Flavobacteriaceae*), and *Planctomycetes* [40]. Only *Betaproteobacteria* were significantly more abundant in the polyps of *Chrysaora* as compared to isolated podocyst. The microbiome of *Chrysaora* clustered according to the life stage rather than by sampling location and time, with polyp bacterial communities being significantly different from their podocyst and excyst. Thus, Lee et al. [40] speculated that the polyp-specific microbiome might be essential for their sessile lifestyle and important for the initiation of later developmental stages. Furthermore, the microbiome of the polyps was more similar to the next developmental stage, the excyst, than to the podocyst, which, as Lee et al. [40] argued, demonstrates an early stage in the successive restructuring of microbial communities from podocysts to polyps. Bacteria that were more abundant in the cyst stages than in polyps included chemolithoautotrophs of the genera *Nitrospira, Nitrospina, Thiogranum*, and *Desulfovermiculus* and nitrogen fixers of the order *Rhizobiales*. It has been suggested that the podocyst capsule harbors a specific beneficial assemblage of microbes to sustain the viability of podocysts [40]. This specific assemblage consists of bacteria affiliated with *Bacteroidetes, Planctomycetes, Alpha-, Beta-*, and *Gammaproteobacteria* and *Chloroflexi*.

One of the most interesting results of Lee et al. [40] was that within the jellyfish-associated microbiome, half of the detected bacteria were closely related to microbial communities found associated with seaweed, sponges, and sea squirts, all of which interestingly have a benthic lifestyle. Bacteria also had closest relatives known to be drivers of major elemental cycles, including methanotrophs. Interestingly, a number of these bacteria were also present in the microbiome of *A. aurita* as determined by Weiland-Brauer et al. [35]. However, it is important to note that for the comparison, bacteria associated with benthic and not medusa stages from the *Aurelia* study should be considered. Whether or not this was taken into account is not clear. Furthermore, Lee et al. [40] speculated that these shared bacteria might play an essential role in the scyphozoan life cycle, especially since the two jellyfish species originate from different marine regions. However, the microbiome of *Chrysaora* exhibited higher diversity than *A. aurita*, potentially explained by the fact that some of the studied life stages of *Aurelia* originated from polyps grown in the laboratory, most likely in an environment with lower microbial diversity than under *in situ* conditions. However, we would like to stress that Lee et al. [40] used 1 µm prefiltered seawater as the medium for their laboratory experiments, which could have introduced ambient seawater bacteria and altered the diversity of the jellyfish-associated microbial community. Also, the studies used different sequencing platforms, sequencing depths, and primers to amplify bacterial 16S rRNA genes, hence comparisons between these two jellyfish species should be done with caution.

Another representative of the *Chrysaora* genus, *Chrysaora hysoscella*, collected in the German Bight was studied in Reference [39]. The pluses and minuses of this study are discussed above (see the paragraph on *Cyanea lamarckii*). For this jellyfish species no significant differences between the microbiomes associated with different body parts were found. The microbiome of the umbrella exhibited the highest variability and richness, while the gonads revealed the lowest richness of all body parts. The microbiomes associated with different life stages indicated a strong selective colonization process, with decreasing diversity from larvae to adult medusae. Unfortunately, the methodological approach used in this study did not provide any insight into the phylogenetic composition of the associated microbiome.

#### 2.1.7. Rhizostomeae

Within Rhizostomeae, *Mastigias papua* (a member of the Mastigiidae family), called spotted or lagoon jellyfish, was studied [41] and a member of the Cepheidae family, *Cotylorhiza tuberculata*, known as fried egg jellyfish, was investigated for its microbiome [42,43].

The first study of the microbiome of Rhizostomeae was on the *Cotylorhiza tuberculata* gastric cavity, adopting a culture-independent molecular approach in combination with classical culturing of aerobic heterotrophs [42]. However, no body compartment or life stage specificity was tested or comparison with the diversity of ambient seawater communities made. Both the culturing and culture-independent approaches revealed similar results: reduced diversity of the gastric cavity-associated microbial community, with four major groups of microorganisms detected. The dominant bacteria were *Spiroplasma*, with the closest relative *S. poulsonii* (*Spiroplasmataceae, Mollicutes*), followed by *Thalassospira* (*Rhodospirillaceae, Alphaproteobacteria*), affiliated with the fish pathogen of the genus *Tenacibaculum* (with the closest relative *T. soleae*), and *Synechococcus*. Cortes-Lara et al. [42] suggested that bacteria affiliated with *Spiroplasma, Thalassospira*, and *Tenacibaculum* might be part of the jellyfish’s digestive system, while *Synechococcus* could have been ingested by *C. tuberculata*. Within the culturable fraction, almost 80% of the bacteria were *Vibrionaceae* (*V. xuii* and *V. harveyi*). However, even though Cortes-Lara et al. [42] reported high similarity of the microbiomes among the studied specimens, one should note that this study was based on only four individuals sampled at just one location in one season. Thus, these conclusions might not be representative of the entire population or the jellyfish species.

A subsequent study [43], also focusing on the gastric microbiome of *C. tuberculata*, this time using a shotgun metagenomic approach, revealed the entire genomic repertoire of the dominant members of the bacterial community. Applying fluorescence *in situ* hybridization using fluorescently labeled oligonucleotide probes to target specific bacterial groups and epifluorescence microscopy (CARD-FISH) allowed for localization and identification of specific bacteria within adult medusae. Viver et al. [43] proposed a simple model of microbial-animal digestive associations and hypothesized on the role of the microbiome of *C. tuberculata* in jellyfish ecology. *C. tuberculata* individuals were collected during the same sampling campaign and location as in the study by Cortes-Lara et al. [42], but over an additional two consecutive years to account for interannual community variability.

One of the most important findings of this study was that the microbiome of the gastric cavity of *C. tuberculata* was dominated by eukaryotic cells (*Onychodromopsis*-like and *Symbiodinium* cells) and their infecting bacteria. In line with the previous study [42], low diversity of the gastric cavity-associated microbial community was found, dominated by a few bacterial species, such as a *Simkania*-like lineage of the phylum *Chlamydia*, *Tenacibaculum*-like, *Spiroplasma*-like, and *Mycoplasma*-like bacteria. The association between the jellyfish and its low-diversity microbiome was temporarily stable and possibly related to food ingestion and protection from pathogens. Microscopic analysis contradicted to some extent the results of the metagenome analysis, emphasizing the importance of coupling microscopy-based methods with omic approaches to study the jellyfish-associated microbiome. Based on their findings, Viver et al. [43] proposed three candidate taxa: *Simkania*-like lineage, *Candidatus* Syngnamydia medusae sp.nov. (affiliated with the candidate genus “*Candidatus* Syngnamydia” [59], in particular *Candidatus* Syngnamydia salmonis [60]), *Spiroplasma*-like bacteria, *Candidatus* Medusoplasma gen.nov, *Candidatus* Medusoplasma mediterranei sp.nov., and *Tenacibaculum*-like bacteria, *Candidatus* Tenacibaculum medusae.

Within Rhizostomeae, the microbiome of *Mastigias cf. papua* was also studied [41]. This study employed 16S rRNA gene 454 pyrosequencing to examine the bacterial community associated with adult medusae collected during three sampling campaigns at three sampling locations during one season. Major groups of the microbiome of *Mastigias cf. papua* were *Gammaproteobacteria* (with *Endozoicimonaceae* being most abundant), followed by *Mollicutes*, *Spirochaetes*, and *Alphaproteobacteria* (with the orders *Kiloniellales* and *Rhodobacterales*).

#### 2.1.8. Cubozoa

The only study that ever made an attempt to investigate the microbiome of Cubozoa was published by Clearly et al. [41] (for study details, see previous paragraph on Mastigiidae family), in particular with *Tripedalia cf. cystophora*. *Endozoicimonaceae* (*Gammaproteobacteria*) were the most abundant microbes associated with the adult medusae, followed by *Spirochaetes, Kiloniellales* (*Alphaproteobacteria*), and the low-abundance *Mollicutes* and *Firmicutes*.

#### 2.1.9. Hydrozoa

Among cnidarians, *Hydrozoa* is the only taxon with freshwater species and formation of colonies combining both polyp-zoids and medusa-zoids; however, one stage or the other, more frequently medusa, is reduced or absent. They are found in nearly every marine habitat type and their diversity is significantly higher than that of Scyphomedusae, the former comprising more than 3800 species. Chronologically, to our knowledge, the first report of bacteria associated with Cnidaria was published in 1978 by Margulis et al. [31], who reported a large number of rod-shaped Gram-negative bacteria with a single polar flagellum, found in gastrodermal cells of healthy freshwater green hydras *Hydra viridis*. Afterward, the microbiota associated with the freshwater *Hydra* was extensively studied (reviewed in Reference [19]). This review focuses on the marine hydromedusan species. Marine Hydrozoa *Phialella quadrata* (*Leptothecata*) and *Muggiaea atlantica* (*Siphonophorae*) were investigated by Ferguson et al. [32] and Fringuelli et al. [34] and recognized as a vector and potential natural reservoir of *Tenacibaculum maritimum*, a bacterial pathogen frequently found in farmed fish. *Tubularia indivisia* (*Anthothecata*) was investigated by Schuett and Doepke ([38]; see first paragraph on Cyaneidae for details of this study), showing that its tentacles are associated with potential pathogenic endobiotic bacteria, such as *Cobetia marina*, *Colwellia aestuarii*, *Endozoiciminas elysicola, Vibrio aestuarianus, Bacillus subtilis*, and *Ilyobacter psychrophilus*. Daley et al. [36] investigated the microbiome of *Nemopsis bachei* (Anthothecata) parallel to the *A. aurita* analysis (see paragraph on Ulmaridae for details of this study). Bacteria found to be associated with *N. bachei* were affiliated with *Gammaproteobacteria* (*Vibrionales, Oceanospirillales, Enterobacteriales*, and *Alteromonadales*), with *Alphaproteobacteria* (*Rickettsiales* and *Rhizobiales*), with *Flavobacteria* (e.g., *Tenacibaculum maritimum*), with *Cyanobacteria* (e.g., *Synechoccous*), and with *Firmicutes*.

#### 2.1.10. Ctenophora

The WoRMS lists over 200 Ctenophora species, but only a few have been examined for their associated microbial communities: among the *Tentaculata* class, *Mnemiopsis leidyi* [26,44,45,61], *Bolinopsis infundibulum* [45], and *Pleurobrachia pileus* (*Cydippida* order) [45], and only one species of the *Nuda* class, *Beroe ovata* [44,45].

Although Ctenophora are known to be parasitized by a variety of eukaryotes, including amoebae, dinoflagellates, and sea anemones [62,63,64,65], probably the first report on bacteria associated with Ctenophora was published by Moss et al. [61]. They observed rod-shaped bacteria in the ciliary structure (g-cilium) of the food grove of the lobate ctenophore *Mnemiopsis mccradyi* (now accepted as *Mnemiopsis leidyi* morphotype) that they described for the first time. Later, bacterial communities associated with *Mneniopsis leidyi* and its natural predator *Beroe ovata* were studied using terminal restriction fragment length polymorphism (T-RFLP), cloning, and sequencing of 16S rRNA genes by Daniels and Breitbart [44]. These authors found that the composition of bacterial communities associated with Ctenophora varied over time (sampling over three consecutive years and different seasons at the same location). *M. leidyi*-associated bacterial communities exhibited some degree of seasonal specificity, with families of *Proteobacteria* and *Bacteroides* being associated with specific months, while no such pattern was observed for *Beroe ovate*-associated bacterial communities. In general, the diversity of the Ctenophora-associated bacterial community was lower than the diversity in ambient seawater assemblages and included some known pathogens of sea anemones. *M. leidyi* was dominated by *Marinomonas* sp. (*Oceanospirillales, Gammaproteobacteria*), previously described in corals and sponges, which were not detected in the water column or in the microbiome of *Beroe ovata*, while the microbiome of *B. ovata* was dominated by *Rhodospirillaceae* (*Alphaproteobacteria*), followed by *Tenacibaculum aiptasiae* a5, originally described as a pathogen of sea anemones, which was not detected in *M. leidyi*. Interestingly, both ctenophores harbored cultured representatives of *Alphaproteobacteria* capable of hydrocarbon degradation, *Thalassospira*, and *Alcanivorax*. *M. leidyi* also contained *Nisaea*, a bacterial genus known to mediate denitrification and nitrite reduction. In both ctenophores, *Mycoplasma* and *Spiroplasma* sp., representatives of the phylum *Tenericutes* were present in low abundance, but were not detected in the surrounding water. Generally, many sequences associated with *M. leidyi* were closely related to bacteria previously described in various marine invertebrates, including corals, sponges, sea anemones, and bivalves, but also some *Betaproteobacteria*, previously reported from freshwater.

Dinasquet et al. [26] investigated the response of bacterioplankton to the presence of *Mnemiopsis leidyi* and analyzed the microbiota of its mesoglea and gastral space. As this analysis supplemented some core experiments and hence was not the main aim of the paper, only one sample of each compartment was analyzed. The resolution of the microbial community composition was high; however, as sequencing of 16S rRNA bacterial genes was performed with next generation sequencing technology 454 pyrosequencing. The gut community was different from the mesoglea community and dominated by *Bacteroidetes* (*Flavobacteriaceae*, with the most dominant bacteria affiliated with *Tenacibaculum*) and *Alphaproteobacteria* (*Rhodobacteraceae*), but also contained *Cyanobacteria* and *Actinobacteria*. Since the examined individuals were starved prior to the analysis, these bacteria could be part of the gut microbiome and were most likely not ingested with prey.

Hao et al. [45] investigated the microbiomes of several species of *Ctenophora*: *M. leidyi, Beroe* sp., *B. infundibulum*, and *Pleurobrachia pileus* collected from Helgoland Roads in the German Bight. Ctenophores were collected three times per week for two consecutive years, 10 individuals of each species each time (in total 496 specimens were analyzed), accounting for seasonal and interannual variability [45]. Total prokaryotic genomic DNA was extracted and analyzed using ARISA and 16S rRNA amplicon pyrosequencing. The four ctenophore species harbored species-specific bacterial communities. The largest difference in microbiome composition was found between *M. leidyi*, a highly invasive species in the region, and the other three species. The seasonal variability in microbiome composition was only analyzed for *M. leidyi*, as it was the only species present throughout all sampling seasons. The *M. leidyi* microbiomes collected in the summer clustered together, clearly separated from the microbiome composition associated with *M. leidyi* collected in autumn and winter. Altogether four major bacterial phyla were detected, *Proteobacteria, Tenericutes, Actinobacteria*, and *Firmicutes*, with *Proteobacteria* being the dominant phylum in all four ctenophore species. While *M. leidyi* and *P. pileus* were dominated by *Gammaproteobacteria*, *Beroe* sp. was dominated by *Alphaproteobacteria*, and *B. infundibulum* harbored roughly equal numbers of *Alpha-* and *Gammaproteobacteria*. The major groups of *Alphaproteobacteria* in *Beroe* sp. were affiliated with *Rhodospirillaceae* (*Thalassospira*). The microbiome of *B. infundibulum* was dominated by *Oceanospirillaceae* (*Marinomonas*), while in *P. pileus*, *Pseudoalteromonadaceae* and *Moraxellaceae* were most abundant. *Beroe* and *M. leidyi* were also associated with a small percentage of *Vibrionaceae*.

### 2.2. Critical Overview of Methodological Approaches Used to Study Jellyfish Microbiome

To depict the characteristics of the microbiome systems of jellyfish, the available datasets and the conclusions of different studies need to be compared. The methodologies used were different, making it occasionally difficult to directly compare the outcomes of these studies. Of particular relevance is that the taxonomic composition of the microbial communities was determined by applying different methods, but also different sampling approaches were used that might affect the relevance of the resulting data. The purpose here is not to criticize individual methodological approaches applied by different studies, since many of them used state-of-the-art techniques at the time they were performed. Rather, we want to point out that direct comparisons frequently cannot be made and general conclusions cannot be drawn when only limited comparable datasets are available.

Considering microscopy-based methods of enumerating microbes, the methodological spectrum spans from light and electron microscopy to epifluorescence and confocal laser scanning microscopy. Early studies applied light and electron microscopy to detect jellyfish-associated microbes and describe their morphology and location within the jellyfish host [31,33,38]. Later, epifluorescence and confocal laser scanning microscopy combined with specific oligonucleotide probes labeled with specific fluorochromes were applied, allowing localization of specific microbial taxa at varying phylogenetic resolutions ranging from kingdom- to strain-specific within specific jellyfish life stages and body compartments [35,43].

The range of techniques available to determine the phylogenetic composition of jellyfish microbiomes spans from culturing to culture-independent molecular biology-based techniques. Classical culturing, mostly applied in early studies, provides insight into only the cultivatable part of the microbial community, which presumably currently represents only 1–5% of the total community [66]. The first culture-independent techniques applied were DNA fingerprinting techniques, such as ARISA, DGGE, and T-RFLP (Table 1). These techniques allow recording of differences in the microbial community structure with varying resolution at low cost, but cover only those populations with a DNA content of 0.1–1% of the total community DNA. These fingerprinting techniques offer only limited possibility to identify the phylogeny of specific members of the microbial community. For example, the DGGE method has high resolution, in terms of detecting 1 bp differences among individual sequences and allows many samples to be analyzed and compared concurrently. However, to obtain information on specific phylogenetic groups of the bacterial community, one has to excise individual DGGE bands—each representing, in theory, different bacterial species/groups and clone and sequence them. In this process, especially the band-excision step is critical, as many bands are not separated well enough, which means that it is not possible to excise some and cross-contamination can occur during the procedure. Clone libraries using the 16S rRNA gene were also widely used as a culture-independent technique to study the phylogenetic composition of individual microbial communities. This approach allowed for in-depth phylogenetic analysis, as the obtained sequences were usually of good quality and long, allowing for classification even to the genus level. This approach, however, does not sufficiently describe the diversity of a given population, as it fails to detect the rare community members. Despite all these limitations and shortcomings, we believe that the early sequencing efforts provided valuable insights into dominant groups of bacteria associated with jellyfish, contributing important information to our understanding of jellyfish microbiomes. Due to these shortcomings and the labor-intensive nature of these methods, next-generation sequencing (NGS) techniques have been applied over the last decade to study the composition of jellyfish microbiomes with high resolution and efficiency. Next generation sequencing approaches have been rapidly developing since they were first introduced to marine microbial ecology by Sogin et al. [67], describing the rare biosphere using 454 pyrosequencing. Currently, the most frequently used NGS platform in microbial ecology is the Illumina MiSeq or HiSeq. Although the resulting data in different studies can be compared most easily, almost all publications on jellyfish microbiome (Table 1) have used different prokaryotic DNA extraction protocols, and different kits to construct DNA libraries, and frequently, different regions of the 16S rRNA gene were amplified with different sets of primers. All of these limits the comparability of the available sequencing datasets.

Nearly all of the studies conducted so far performed only bacterial 16S rRNA gene amplicon sequencing to obtain information on the phylogenetic diversity of the jellyfish microbiome. Therefore, hypotheses on the function and role of the microbiome associated with jellyfish and the relationships and interactions within host–microbiome associations are merely speculations based on our knowledge of specific microorganisms. The one exception to this is the study on the gastric cavity of *Cotylorhiza tuberculata*, in which a metagenome approach was used providing insight into the genetic potential of the microbiome [43]. To our knowledge no study on the metatranscriptome, metaproteome, or metabolome level of the jellyfish microbiome has been performed so far.

Studies have analyzed the jellyfish microbiome at different levels, investigating the degree of microbiome specialization, species specificity of the microbiome, jellyfish life stage and body compartment specificity, and jellyfish population specificity (Table 1). However, to our knowledge, a holistic approach covering all possible aspects in a single study has never been used. For example, the degree of specialization was addressed only occasionally by comparing the degree of similarity between the jellyfish microbiome and ambient water microbial communities [26,35,36,41,44]. Concurrent analyses of the jellyfish microbiome and the host’s natural background, i.e., the pool of ambient water microbial community, would also provide insights into possible transmission mechanisms of the jellyfish microbiome (i.e., horizontal vs. vertical transmission).

Another important point to consider is the fact that most of the studies examined different jellyfish species collected from diverse marine systems, where jellyfish are continuously present or occur seasonally. A comparison of microbiomes associated with the same jellyfish species collected from different marine systems would enable us to understand the population specificity of the associated microbiome. However, this comparison is currently only possible for a few jellyfish families, i.e., Cyaneidae, Pelagiidae, Phialellidae, and Bolinopsidae, and in one single case at the genus level, i.e., for *Aurelia aurita* (Table 1). In addition, there are several studies on different jellyfish species from the same system, which allow us to speculate on the effect of the microbial community of the ambient water on the jellyfish microbiome [34,36,38,39,41,44,45]. The spatial patchiness and variability within specific systems have rarely been addressed [40,43], as mostly jellyfish collected from a single sampling location were analyzed. Studies have rarely accounted for the variability within jellyfish populations, as most are based on only a small number of specimens. Furthermore, only few studies have accounted for the interannual [40,43] and seasonal [37,44,45] variability of jellyfish–microbe associations. With a single exception [42,43], no studies of specific jellyfish species from the same marine system are available where different groups of researchers have studied them. Consequently, no critical assessment of the reproducibility of the results can be made. Most of the studies were performed on jellyfish collected from their natural habitat, usually during bloom conditions, but some studies were conducted using jellyfish grown in the laboratory [35,40].

Jellyfish were collected and analyzed at different stages of their life cycle, but this comprehensive approach was applied only rarely [35,39,40]. To our knowledge, there is just a single study that investigated the microbial community associated with each life stage of a specific jellyfish (*Aurelia aurita*, [35]). However, even in this case, not all body compartments were analyzed, and in the future, the methodological approach should be improved to avoid possible cross-contamination.

## 3. Characteristics of the Jellyfish-Associated Microbiome

The screening and critical overview of all published studies to date on the jellyfish-associated microbiome revealed some potential characteristics and patterns of jellyfish-associated microbial communities.

### 3.1. What is the Degree of Specialization of the Jellyfish-Associated Microbiome?

Only a few attempts have been made to investigate whether there is a jellyfish-specific microbiome. Are these microorganism generalists and thus also present in the water column or on organic detrital particles? Are they generalist symbionts (i.e., can be found in association with other similar organisms) or specialists (i.e., are found only in association with jellyfish)?

The approach applied by these studies was to compare the diversity of the jellyfish-associated microbiome with the bacterial community in the ambient water. The conclusion from these studies is that the jellyfish-associated bacterial community is significantly different in terms of composition and lower in diversity than the bacterial community in the ambient water. This was reported in all the studies on *A. aurita*’s microbiome from the North Atlantic coastal waters [36], Kiel Bight, Baltic Sea, English Channel, North Sea [35], and Northern Adriatic Sea [37]. In addition, this was also reported for the ctenophore *M. leidyi*, although only in one study so far [44].

The jellyfish-associated microbiome is frequently also found associated with other host organisms, such as corals, sponges, sea squirts, and sea horses, which sometimes also share similar lifestyle, morphological, and/or biochemical characteristics [37,40,41]. This indicates a certain degree of specialization of the jellyfish-associated microbiome, suggesting that the jellyfish microbiota could be symbiotic generalists. Some studies concluded that at least certain members of the jellyfish-associated microbiome are specific for certain jellyfish taxa. For example, a comparison of the microbiomes of *Chrysaora* and *Aurelia* revealed a certain number of shared bacterial species, suggesting that they are specific for scyphozoans in general [40]. However, one should be careful when drawing general conclusions from a limited number of investigations performed with different methods. There is evidence, however, that some members of jellyfish-associated microbiomes are rarely present in ambient water microbial assemblages and are more frequently found in association with similar organisms, probably due to their ability to attach and thrive under the specific environmental conditions (e.g., high viscosity, high nutrient, and low oxygen concentrations, presence of toxins and other antimicrobial compounds) that these organisms have to offer.

The observed low-diversity microbiome of jellyfish is in contrast to the trend observed for corals and sponges, where the diversity of associated microbiomes is usually higher than that in the ambient water [11,68,69,70]. One possible explanation is the production of antimicrobial compounds by jellyfish or their associated microbiomes. As discussed by Cleary et al. [41], *A. aurita* produces an antimicrobial peptide, named aurelin, that is active against Gram-positive and -negative bacteria [71]. Likewise, extracts of *Cassiopeia* spp. showed strong antimicrobial activity against bacteria [72]. Antibacterial polyketides were also isolated from a fungal symbiont (*Paecilomyces variotii*) of the jellyfish *Nemopilema nomurai* [73]. Thus, survival in this specific environment might be possible for only some bacterial taxa, leading to reduced diversity within the jellyfish-associated microbiome. In conclusion, the degree of specialization of the jellyfish-associated microbiome needs to be further studied, expanding to different taxonomic groups of jellyfish, marine systems, and biogeographic provinces.

### 3.2. Is the Jellyfish-Associated Microbiome Jellyfish Population-Specific?

The fact that studied jellyfish were collected in different marine systems ranging from the North Atlantic Ocean to marine lakes in Indonesia (Table 1) might help to determine whether the microbiome is specific to certain jellyfish populations.

Jellyfish are known to be able to inhabit very different marine systems around the world, from shallow coastal seas to deep-sea environments, and, even more, seem to be able to quickly acclimatize to changing physical conditions (e.g., temperature, salinity [74,75]) and adapt to emerging features of marine habitats such as lack of predators/competitors caused by overfishing [76] or use of increasing marine sprawl, such as platforms as a surface for their polyp phase [77]. This could also be reflected in their associated microbiome. From the research conducted so far, the question of whether there is population specificity of the microbiome can only be addressed for *A. aurita*, as it was analyzed from different marine systems (North Atlantic [36], Kiel Bight, Baltic Sea, Southern English Channel, North Sea [35], and Northern Adriatic Sea [37]), and for *M. leidyi* from its native environment, Tampa Bay (Florida, USA) [44], and to areas where it was recently introduced, such as Gullmar fjord (Sweden) [26] and Helgoland Roads (German Bight) [45]. Interestingly, the results for the two jellyfish phyla contrast. Whereas in scyphozoan *A. aurita* the microbiome seems to be population-specific and hence varies with sample location, the ctenophore *M. leidyi* harbored a similar microbiota regardless of the sample location (e.g., *Marinomonas* was detected in ctenophore from all studied systems).

If we assume that the jellyfish-associated microbiome is determined to some extent by the environment, many more jellyfish ecosystems should be explored to better understand the selective pressure. In particular, one would expect differences in the microbiome of open-ocean (or even deep-sea) vs. coastal jellyfish, which are subjected to different degrees of anthropogenic impact. In particular, coastal environments are common entry points of pathogenic bacteria into marine ecosystems via coastal runoff, wastewater treatment plant discharge, or other kinds of human activities. Furthermore, as pathogenic bacteria are known to prefer organic-rich environments and are capable of surface attachment, they could easily hitchhike with bypassing jellyfish or, even more likely, attach to and thrive on polyps commonly found on biofouling at pillars in ports, on platforms, or on other structures. As known filter feeders, some of the organisms that dominate in biofouling communities, such as mussels and oysters, can accumulate pathogens and microbes that produce toxic compounds, which could be transmitted to polyps and from there to adult medusae, which can drift large distances. In this way, jellyfish can be seen as vectors for pathogens and other allochthonous bacteria from coastal to open water. This could be important in light of the recently modelled dynamics of jellyfish populations showing that a population of jellyfish can drift far away from its source polyp population area [77]. Another important unexplored aspect of the transmission route of the jellyfish microbiome is ballast water, potentially introducing invasive/nonnative species of both jellyfish and microbiota into new marine environments.

### 3.3. Is there a Jellyfish Taxa-Specific Microbiome?

Several studies have compared the composition of microbial communities associated with different jellyfish taxa collected from the same environment (Table 1). In a study on an Indonesian marine lake, representatives from different medusozoan classes were collected, i.e., a jellyfish from the Mastigiidae family, representative of the Scyphozoa class, and a jellyfish from the Tripedaliidae family, representative of the Cubozoa class [41]. This study indicated that the microbiome is jellyfish taxa-specific [41]. Another study performed on scyphozoan jellyfish of the order Semaeostomeae, *Cyanea lamarckii*, a representative of Cyaneidae family, and *Chrysaora hysoscella*, a representative of Pelagiidae family, both collected in the German Bight, showed that there was a clear difference between the microbiome associated with the life stages of the two species, indicating specificity of the microbiome for certain jellyfish taxa [39]. In a study on two species of Cyaneidae, *Cyanea capillata* and *Cyanea lamarckii*, collected in Scottish coastal waters, their microbiomes were found to be species-specific [38].

### 3.4. Is the Jellyfish-Associated Microbiome Specific to Different Life Stages?

Selecting jellyfish as a host seems to be an advantage or survival strategy for at least some bacteria. However, due to dramatic changes of lifestyle in the life cycle of jellyfish (benthic/attached vs. pelagic/swimming), morphology (i.e., surface architecture), and (bio)chemical characteristics (i.e., different expression patterns of antimicrobial compounds during different life stages), its associated microbiome needs both resiliency and plasticity. Each life stage might represent a unique niche allowing for specific bacteria to grow, and consequently, life stage specificity of the microbiome might be possible. From a jellyfish perspective, different life stages are associated with different necessities and requirements, which could drive corresponding shifts in the structure of associated microbiomes, as hypothesized by Lee et al. [40].

Microbial communities associated with different life stages of specific jellyfish species have been only rarely investigated [35,39,40]. Comparing the results of these studies, it seems that the composition of the jellyfish-associated microbiome changes with the life stage, particularly, during the transition from the benthic to the pelagic stages. The observed shifts in the community composition during the different developmental stages raise questions on the functional role of the microbiome in general and the development of jellyfish, as discussed by Weiland-Brauer et al. [35]. Certain members of the microbiome might have specific metabolic functions, which might play a role during specific development stages of the jellyfish [35]. However, available data on the diversity of the microbiome associated with different jellyfish life stages provide information only on the genetic potential or potential metabolism of specific bacterial groups. Hence, one can only speculate on the functions of specific bacteria in different life stages of jellyfish.

Our current knowledge of the bacterial colonization of scyphozoans or other jellyfish larvae, the establishment of the microbiome, and the compositional stability at different life stages, especially during strobilation, is limited. In jellyfish, however, bacteria have been shown to be important for larval settlement: it was shown that the settlement of pedal stolons of scyphopolyps of *A. aurita* was induced by *Micrococcaceae*, presumably via its effective substances acylgalactosidyldiglyceride and monogalactosidyldiglyceride [78,79], while in *Cassiopea andromeda*, swimming buds and planulae were induced to settle and metamorphose by a compound released from a *Vibrio* species during growth [80,81,82,83]. Regulation of the jellyfish life cycle, in particular the transition from one life stage to another and the induction of metamorphosis, is still only poorly understood. Recently, three major studies shed light on the regulatory mechanisms of the life cycle of jellyfish. One study focused on transcriptome profiling of the life stages of *A. aurita* [84], another study focused specifically on molecules critical in controlling the polyp-to-jellyfish transition [85], and the third study determined the genome of *A. aurita* [86], representing the first fully sequenced genome of the medusa stage of a cnidarian and providing important insight into the evolution of animal complexity. These analyses of life stage transcriptomes showed that different transcript expression profiles are related to each specific life stage and that not only shifts in ambient temperature, but also other signaling factors could initiate and regulate the strobilation and metamorphosis processes. It was shown that the nuclear hormone receptors, including the retinoic acid signaling cascade, are core elements of the regulation machinery of the life cycle of *A. aurita*, identifying strobilation inducer and precursor, novel CL390 protein, and its minimal pharmacophore, 5-methoxy-2-methylindole [85]. We argue that additional strobilation regulators might be present, and it is tempting to speculate on the role that ambient or associated microbes play in this process, especially as this was not tested in any of the studies conducted so far and the exclusive production of these compounds by the host has not been shown. One might speculate that similar or antagonistic molecules are produced by the jellyfish-associated microbiome in particular, since it was shown that bacteria by themselves or via their extracellular vesicles induce metamorphosis in marine invertebrates ([87] and references therein). The identification of strobilation and proliferation agents also has direct biotechnological application, as it might represent an avenue to control jellyfish blooms [85].

In addition, it was speculated that for the benthic life stage, the associated microbiome is particularly important for producing vitamins, amino acids, and secondary metabolites and possibly for inducing subsequent developmental stages [35]. Among bacteria associated with *A. aurita* polyps, *Phaeobacter* is known as a producer of compounds that inhibit fouling of surfaces of the host and substrates in general by interfering with cues for the settlement of invertebrate larvae and spores of algae [88,89,90]. Also, the polyp-associated bacteria of the genus *Rhodococcus*, the only exclusively marine genus within *Actinobacteria*, produce extracellular enzymes and are an exceptionally rich source of secondary metabolites, particularly compounds with the potential to serve as novel antibiotics and anticancer drugs (reviewed in Reference [91]). Polyp-associated bacteria of the genus *Vibrio* are known to produce quorum-sensing signals, and their antagonistic behavior was proposed to stimulate the settlement of spores of other organisms [88,92]. In a study of *Chrysaora plocamia*, polyps were proposed to form the base for a plethora of asexual reproduction strategies, and it is likely that they represent a reservoir of microbial members essential for the initiation, development, and survival of the subsequent life forms [40], while the authors hypothesized that the microbiota associated with *C. plocamia* podocyst are conserved within the podocyst capsule, where they help to sustain the viability of podocysts and potentially facilitate the activation of excystment [40].

Taking all this information together, it seems that a major restructuring of the polyp-associated microbiome takes place at the transition from the benthic to the pelagic stage, also in terms of reduced diversity. Different pelagic stages seem to be more similar to each other than to the benthic stage. The observed changes could be a response to different lifestyles, metabolisms, morphologies and biochemical characteristics of jellyfish, but could also be due to different environmental conditions that the microbiome experiences in the benthic vs. pelagic stage of the host.

### 3.5. Is the Jellyfish-Associated Microbiome Body Part-Specific?

In jellyfish, different types of cells are present in different body compartments [93]. Body compartments are characterized by different morphological and biochemical features, as well as by their degree of contact with the ambient water. Thus, different body compartments might be preferentially colonized by specific microbes.

Few studies have investigated the compartment specificity of the jellyfish microbiome. In *A. aurita*, the differences between the microbiomes of the mucus and the gastric cavity were investigated [35]; in *Cyanea lamarckii* and *Chrysaora hysoscella*, the composition of the microbiomes of the tentacles, umbrella, mouth arm, and gonads was studied [39]; and in ctenophore *M. leidyi*, differences in the microbiome composition between the gut and tissue were investigated [26]. In other studies, jellyfish were analyzed as whole individuals, even when they were dissected prior to analysis (Table 1).

#### 3.5.1. Microbiome Associated with Outer Body Parts and Its Potential Role

For all investigated species, compartment specificity of the jellyfish-associated microbiome has been reported, with outer body parts, e.g., umbrella and mucus, usually harboring more diverse, rich, and variable microbial communities than the inner body compartments, e.g., gonads and gastric cavity [26,35,37,39]. The degree of similarity between the microbiomes associated with outer body compartments and ambient water bacterial assemblages was higher than that between the microbiomes of inner body compartments. The jellyfish’s outer body compartments are in direct contact with microbial communities of the ambient water, thus the microbiome of the outer compartments is probably directly recruited from those communities, indicating the possibility of horizontal transfer of the jellyfish’s ecto-microbiome.

The epidermis and gastrodermis of the jellyfish contain numerous types of unicellular mucus-producing gland cells, leading to the formation of a thin, constantly renewed mucus layer covering the entire external surface of the medusa [94,95]. Under conditions such as stress and moribundity, but also during reproduction and digestion, mucus release rates are higher than under nonstress conditions [95]. Mucus contains toxins and nematocysts, thus serves as an important chemical defense mechanism of jellyfish and plays a major role in surface cleansing [95,96]. Jellyfish also produce toxins and antimicrobial compounds, such as the peptide aurelin in the mesoglea of *A. aurita* [71]. The mesoglea is an extracellular matrix situated between the epidermal and gastrodermal layers [97] containing collagen and collagen-like proteins associated with mucopolysaccharides [98,99]. Secreted mucus and the mesoglea are mainly composed of proteins, lipids, and, to a lesser extent, carbohydrates in different ratios [100,101], representing an attractive niche for bacteria, especially those with a competitive advantage and specialized for settling from the ambient water. The specific physiochemical characteristics of mucus and the physiology of the host represent selective pressure and determine the abundance and diversity of metabolically active bacteria [102]. Hence, one can hypothesize that jellyfish can actively or passively select their bacterial associates. Whether bacteria directly adhere to external cell layers of jellyfish or are only associated with the thin mucus layer remains to be resolved.

Based on the two studies that actually focused on the composition of the microbiome associated with jellyfish mucus, *Gammaproteobacteria*, particularly *Pseudoalteromonas* and *Vibrio*, are abundant, but to some extent also *Alphaproteobacteria* (*Phaeobacter*, *Rugeria*, and *Roseovarius*) [35,37]. These bacteria were previously recognized as important players in the host defense against pathogens and fouling organisms from the surrounding seawater [88,92,103,104] because of their ability to produce antimicrobial compounds when attached to live or inert surfaces [90,104,105,106,107]. Considering that the host would recruit microbes that are beneficial for its development or contribute to its well-being [92], one might speculate that the mucus-associated microbiome serves as a defense mechanism to protect the jellyfish from hostile microbes and other organisms in the ambient water. It was even proposed that the antimicrobial compounds and even toxins found in jellyfish mucus could originate from the associated microbiome (by Schuett and Doepke [38], who found tetrodotoxin-producing bacteria on jellyfish tentacles). At the same time, the mucus-inhabiting microbiome could benefit from constant nutrient input and/or other compounds from the host.

#### 3.5.2. Microbiome Associated with Inner Body Compartments and Its Potential Role

The inner body parts of jellyfish, such as the gonads and gastric cavity, are isolated from the surrounding environment and have different morphological and biochemical characteristics, allowing for development of very specific bacterial groups that are otherwise rarely found in the ambient water. This is in line with reports on lower diversity, richness, and variability of the microbiome of the inner body compartments of jellyfish [26,35,37,39]. Reduced microbial diversity has been found in the gastric cavity of *Cotylorhiza tuberculata* [42,43]. Since a considerable fraction of the bacterial community present in the inner compartments of jellyfish is rarely or never found in the ambient water, these bacteria may have lost the ability to live independently and may be acquired by the host via vertical transmission as parental heritage through reproductive cells and larvae [43]. Based on metagenomic analysis, the role of these bacteria is possibly related to food digestion and protection from pathogens [43].

### 3.6. The Composition, Potential Role, and Biotechnological Potential of the Jellyfish-Associated Microbiome

Overall, it is not surprising that the jellyfish-associated microbiome is a consortium of bacteria (see below) that prefer a particle-attached lifestyle, are capable of degrading complex organic compounds, and are known to be found in association with other marine organisms. These bacteria are known for commensalism, symbiosis, and parasitism, or are even pathogens of marine organisms. Frequently, they are capable of producing quorum-sensing signal molecules, antagonistic compounds, and/or factors that interfere with quorum sensing of other microbes (which involves sensing the abundance of other bacteria, expressing of virulence factors, and interfering with the chemical communication of other bacteria, exhibiting antagonistic behavior). All of these features have either a direct or indirect application in blue biotechnology (Table 2). However, so far there has been no comprehensive study investigating the biotechnological potential of bacteria associated with jellyfish. Therefore, the listed features (Table 2) are based on the literature on the biotechnological potential of these bacterial strains or their closest relatives isolated from other marine organisms or substrates. Bacteria associated with jellyfish are also known for their diverse metabolisms and involvement in the cycling of carbon, nitrogen, sulfur, and phosphorus, which prompted several authors to speculate on their role in supplying specific compounds to jellyfish, implying that there is a symbiotic relationship between jellyfish and the associated microbiome (discussed in detail below). For example, it was proposed that nitrifying bacteria could play an important role in the life cycle of jellyfish [40], as they are known to harbor key enzymes involved in the conversion of ammonia to hydroxylamine and further to nitric oxide, the latter known as an important messenger molecule to regulate metamorphosis in marine invertebrates [108], regulate swimming of the jellyfish *Aglantha digitale* [109], and facilitate the discharge of nematocytes in the sea anemone *Aiptasia diaphana* [110]. All of these features have the potential to be exploited by blue biotechnology (Table 2). Several jellyfish-associated bacteria were previously also associated with the processing of more peculiar substances, such as PAHs, plastics, and xenobiotics found in the ocean, with possible benefits for the host [37,40]. For example, the presence of PAH-degrading bacteria agrees with the high tolerance of *A. aurita* to crude oil exposure and its ability to accumulate PAHs [111], suggesting that the PAH-degrading microbial community associated with *Aurelia* facilitates the survival of jellyfish in polluted coastal systems. This also agrees with the findings of Kos Kramar et al. [37], who detected PAH and plastic-degrading bacteria within the gastric cavity of *A. aurita* collected in the Northern Adriatic. The potential of these microbes and/or the compounds they produce to be used for biotechnological applications is obvious (Table 2). Marine archaea are known for their biotechnological potential (reviewed in References [112,113]); however, only one study so far made an attempt to investigate the archaeal community potentially associated with jellyfish [42]. However, amplification of archaeal 16S rRNA failed [42].

The most frequently detected members of the jellyfish microbiome are affiliated with the following representatives: *Alpha-* and *Gammaproteobacteria*, *Bacteroidetes*, *Tenericutes*, and *Cyanobacteria*. Among the less frequently, but still repeatedly reported were bacteria affiliated with *Betaproteobacteria*, *Spirochaetes*, *Actinobacteria*, *Firmicutes*, *Chlamyidiae*, *Chloroflexi*, *Planctomycetes*, *Nitrospirae*, and *Nitrospinae*. As we have shown that the associated microbiome is probably not exclusively associated with jellyfish and is to some extent affected by the physiochemical boundary conditions, the term “transient microorganisms” would probably best describe the jellyfish-associated microbiome [114].

#### 3.6.1. Gamma- and Alphaproteobacteria

Within the *Proteobacteria*, *Gammaproteobacteria* are reported to be associated with every comprehensively studied jellyfish taxon so far, where they either dominated or at least represented a very substantial part of the jellyfish-associated microbiome. The exception to this seems to be the jellyfish gut microbiome, where either only a small fraction of *Gammaproteobacteria* was detected [42,43] or none at all [26]. Within *Gammaproteobacteria*, different families were regularly reported as a part of jellyfish-associated microbiome: *Vibrionaceae*, *Pseudoalteromonadaceae*, *Alteromonadaceae*, *Oceanospirillaceae*, *Shewanellaceae*, *Crenotrichaceae*, *Methylococcalaceae*, *Endozoicimonadaceae*, *Moraxellaceae*, *Xanthomonadaceae*, and *Legionelaceae*. In general, *Gammaproteobacteria* in marine environments are associated with the ability to attach to surfaces and to degrade high-molecular-weight compounds. Within this class, there are many readily culturable bacteria, such as *Vibrio*, *Alteromonas*, *Pseudoalteromonas*, *Marinomonas*, *Shewanella*, and *Oceanospirillum* [115]. This suggests that most of the jellyfish-associated *Gammaproteobacteria* could be readily cultured, facilitating their possible exploitation for biotechnological applications (Table 2). Furthermore, these bacteria usually exhibit rapid growth and a feast-or-famine lifestyle with quorum-sensing playing an important role. In addition, many of these bacteria are found in biofilms and/or in association with other marine organisms (as symbionts or pathogens) and occupy micro-niches with specific environmental conditions (i.e., temperature, oxygen, nutrient availability). All of the listed features have direct and/or indirect potential for application in biotechnology (Table 2). For example, *Marinomonas* was detected as an abundant member of the microbiome of the ctenophore *M. leidyi* in several marine systems. In terms of their biotechnological potential, these bacteria contain multifunctional polyphenol oxidases that are able to oxidize a wide range of substrates, are producers of antibacterial compounds, and are involved in biodegradation processes [116,117].

*Marinomonas* also contain genes for the breakdown of dimethylsulfoniopropionate, indicative of their role in the cycling of sulfur [44]. *Pseudoalteromonas* is known to produce a variety of highly bioactive compounds, including extracellular enzymes, exopolysaccharides, and compounds involved in antimicrobial antifouling, with algicidal activity and various pharmaceutically relevant activities [118]. *Vibrio* is known as a producer of quorum-sensing signals and for its antagonistic behavior, but also for its proposed role in stimulating the settlement of spores of other organisms [119].

Within the jellyfish-associated *Alphaproteobacteria*, representatives of the *Rhodospirillaceae*, *Rhodobacteriaceae*, and *Kiloniellaceae* families and the order *Rhizobiales* are documented (Table 2). Among them, members of *Rhodobacteriaceae* can easily be cultivated and are commonly found in association with living organisms and detrital particles, in sediment and microbial mats, playing an important role in carbon and sulfur cycles. The *Phaeobacter* genus of the *Rhodobacteriaceae* family is known as a producer of inhibitory compounds that prevent or inhibit fouling of surfaces by interfering with cues for the settlement of invertebrate larvae or spores of algae [88,89,90], a feature with potential for biotechnological application. One of the most reported *Rhodospirillaceae* associated with jellyfish are bacteria affiliated with *Thalassospira* [42,44,45]. Bacteria of the *Thalassospira* genus might be involved in carbon cycling by providing an additional source of fixed carbon for jellyfish and exhibit chemotaxis to phosphate (as suggested in Reference [44]). Bacteria from this genus were found in microbial consortia degrading aromatic hydrocarbons [120,121] as part of the microbiome of sabellids (Polychaeta, Annelida) in crude oil enrichments with potential production of biosurfactants [122], features with biotechnological potential. Bacteria of the *Kiloniellaceae* family are also producers of antibiotic compounds [123], with direct application in biotechnology.

#### 3.6.2. Bacteroidetes, Flavobacteria, Flavobacteriaceae

Jellyfish-associated bacteria of the *Bacteroidetes* phylum are affiliated with the *Flavobacteriaceae* family, and were found in association with Medusozoa, within which with Semaeostomeae, in particular with Ulmaridae (*A. aurita*, [35,36]), with Pelagiidae (*P. noctiluca* and *C. plocamia*, [33,40]), with Rhizostomeae (*C. tuberculata*, [42,43]), and with Ctenophora, where they were not always detected in the same species, suggesting that their presence within the ctenophore microbiome might depend on food and/or the environment [26,44,45]. *Flavobacteriaceae* were also detected in all studied Hydrozoa from different systems. Therefore, *Flavobacteria* represent an important part of the jellyfish-associated microbiome, as they are widespread within jellyfish. However, the association seems to be dependent on the host’s natural environment to some degree. This bacterial group is known to be easily cultivated and its most distinctive properties are gliding motility and the expression of various extracellular hydrolytic enzymes to degrade complex organic materials, with potential application in biotechnology (Table 2). Some members are pathogenic and some psychrophilic, both characteristics with biotechnological potential. They can be found in the human gut and, in sewage-polluted waters, but also in seawater, where they persist for a long time, thus were proposed as indicators of water quality [124], also with potential biotechnological application. Among the most frequently reported members of the *Flavobacteria* found in association with different jellyfish species are bacteria affiliated with the genus *Tenacibaculum*. *Tenacibaculum maritimum* is a known fish pathogen that causes tenacibaculosis, a disease considered to be an important threat to aquaculture worldwide [125]. *Tenacibaculum maritimum* was the first specific bacterium associated with jellyfish to be extensively studied, after the first report that it might infect fish gills damaged by jellyfish venom [126]. Consequently, 20 years ago, jellyfish, in particular *Phialella quadrata*, *Cyanea capillata*, and *Pelagia noctiluca*, were recognized as a possible cause of mass mortality of fish farmed in sea cages [30,127,128]. Transmission of this bacterium via the ambient water and direct transmission from host to host have been proposed as possible routes of infection, in addition to ingestion through food [129]. However, as the survival of *T. maritimum* in seawater is rather limited [130], its natural reservoir remains unclear. Ferguson et al. [32] provided the first evidence of *Phialella quadrata* carrying filamentous bacteria affiliated with *T. maritimum* and proposed that jellyfish is a vector and a carrier of this fish pathogen. These authors suggested that the presence of this proteolytic enzyme-producing bacterium in the jellyfish mouth could support the pre-digestion of the prey of jellyfish and that it could be specific to jellyfish, playing an important role in both immune defense and their nutrition [32]. In a subsequent study, the presence of *T. maritimum* in the mouth of *Pelagia noctiluca* was shown [33]. Supported by the results of both studies, Delannoy et al. [33] proposed that some cnidarians might represent a natural host for *T. maritimum*. However, the environmental reservoir of *T. maritimum* has not been determined yet. Also, its survival in seawater and the suitable niche for its growth in ambient water remain unclear. Quantitative real-time PCR was applied to detect *T. maritimum* in both of the tested species, *Phialella quadrata* and *Muggiaea atlantica* [34]. Bacteria affiliated with the genus *Tenacibaculum* were also found in association with *Cotylorhiza tuberculata* [42,43], with hydromedusa (*N. bachei* [36]), and with the ctenophores *M. leidyi* [26] and *B. ovata* [44]. Based on the large number of genes indicative of carbohydrate and protein metabolism, it has been suggested that *Tenacibaculum*-like bacteria are polymer degraders in jellyfish and their high abundance in the mesogleal axis of the gastric filaments indicate their role in the digestion of ingested food items such as copepods [43]. The possibility for the biotechnological application of this bacterial species as a diagnostic tool and beyond is obvious (Table 2).

#### 3.6.3. Tenericutes

Within the *Tenericutes* phylum, the class *Mollicutes* has frequently been associated with jellyfish. In particularly, in *A. aurita* it has been suggested that *Mollicutes* is a potential endosymbiont [35,36]. In addition, *Mollicutes* was detected in both studied representatives of Rhizostomeae, in the gastric cavity of *Cotylorhiza tuberculata* [34,43] and associated with the Mastiigiidae family. *Mollicutes* was also reported as part of the microbiome of Cubozoa [41] and frequently associated with ctenophores [44,45]. Most of the detected bacteria within the *Mollicutes* class were affiliated with either the *Spiroplasmataceae* or *Mycoplasmataceae* family. The unique characteristics of *Mollicutes* are the lack of a cell wall, small size and simple cell structure, reduced genome, and simplified metabolic pathways, all indicative of a parasitic lifestyle corresponding to their preferred habitat in jellyfish, the gastric cavity, and their suggested endosymbiotic relationship with jellyfish. These bacteria are widespread commensals or pathogens of humans, mammals, reptiles, fish, plants, and arthropods and have been reported also in cnidarians, such as corals [131,132], and are known for their antimicrobial resistance, all characteristic with the potential to be exploited for biotechnology. Their frequent detection as part of the jellyfish microbiome indicates that they are important members of the jellyfish-associated microbial consortium. However, it is important to note that these microorganisms are also one of the most common sources of cross-contamination in academic and biopharmaceutical production laboratories. For the *Spiroplasma*-like bacteria identified as the dominant microbes in the gastric cavity of *C. tuberculata*, the estimated genome was smaller than any other currently known genome of *Spiroplasmas*, which might be indicative of their intracellular lifestyle as predicted anaerobic fermenters. The apparently fit status of the analyzed jellyfish suggests that the intracellular *Spiroplasma*-like bacteria are commensals of *C. tuberculata* rather than pathogens [43]. Features of these bacteria with biotechnological potential are summarized in Table 2; however, the full biotechnological potential of *Mollicutes* associated with jellyfish remains to be explored as a major problem in research with these bacteria is the difficulty of cultivating them in vitro.

#### 3.6.4. Minor Members of the Jellyfish Microbiome

*Betaproteobacteria* that were detected as a part of jellyfish microbiome affiliated with *Burkholderia*, *Achromobacter*, and *Cupriavidus* [37,40,44] were previously associated with degradation of PAHs, plastics, and xenobiotics in the marine environment [88,89,90]. Direct application of these features in biotechnology is apparent (Table 2). *Spirochaetes* found in association with Rhizostomeae, in particular with the Mastigiidae family, and with Cubozoa are known for their unique morphology, with tightly coiled spirals, and their motility. They are very widespread in marine habitats, but not much is known about their ecological role or their biotechnological potential. However, they might play an important role in the gut microbiota of marine animals. *Spirochaetes* were reported in two jellyfish species in one stud, in which both species were collected at the same sampling location, an Indonesian marine lake [41]. This raises questions about a location-specific pattern and the methodological approach used by this particular study [41]. In any case, these bacteria include aerobic and anaerobic species, well-known pathogens (syphilis and Lyme disease), and mutualists, for example, inhabiting the guts of cows and termites ([41] and references therein), all features with biotechnological potential. Also, *Actinobacteria* were not common in the jellyfish microbiome; however, they were found in association with *A. aurita* [35,37] and with *M. leidyi* [26,45]. *Actinobacteria* is a bacterial taxon with the greatest biotechnological potential known [133]. *Firmicutes* probably represents a minor part of the jellyfish microbiome as well. However, since they are otherwise rarely recorded as part of natural marine microbial assemblages, their presence within the jellyfish microbiome might indicate that they play an important role in this consortium. They were recorded in association with Cubozoa [41] and with different ctenophores [45]. The biotechnological applicability of these bacteria (e.g., in remediation of polluted marine sediments, as probiotics in aquaculture) and their features (e.g., pathogenicity, resistance to high temperatures, irradiation, desiccation, wide range of fermentation pathways, source of toxins) are known (reviewed in Reference [6]). Within *Chlamydiae*, *Simkania*-like bacteria were found to be the most dominant microorganism in the gastric cavity of *C. tuberculata*. Based on their metagenome, Viver et al. [43] speculated that they are putative endosymbionts of a ciliate that was probably symbiotic with the jellyfish. These authors speculated that the ciliate might be involved in controlling the free-living microbial population within the gastric cavity through grazing, therefore reducing competition between the host and the specific bacterial population for food (i.e., copepods). Furthermore, the authors speculated that the role of the *Simkania*-like endosymbiont of a ciliate, for which metabolic modelling predicts an aerobic heterotrophic lifestyle, could be understood as a nested symbiosis (supported by size and genetic repertoire exhibiting a versatile lifestyle) or even potential pathogenic capability (supported by the repertoire of genes indicative of virulence factors, and their relationship could be mutually beneficial for the host), features with biotechnological potential.

## 4. Conclusions and Future Research Directions

**Figure 1:**
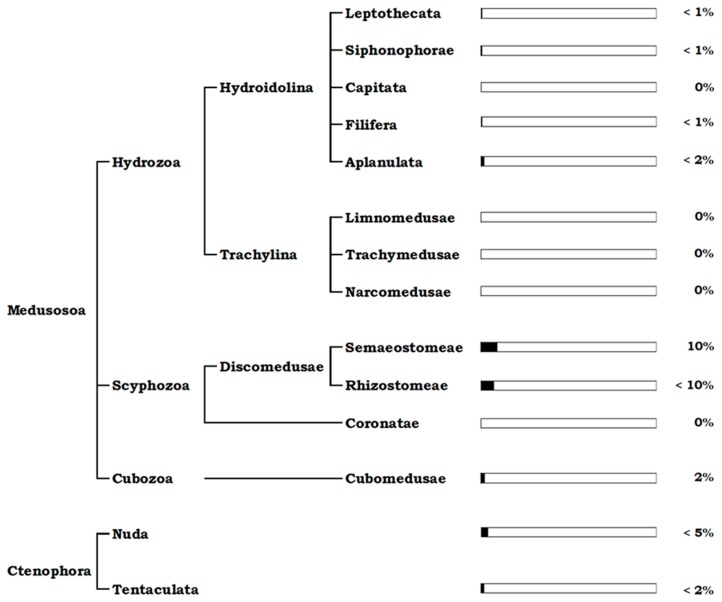
Relative amounts of investigated species in gelatinous taxa. Number of species per taxon was assembled from World Register of Marine Species (WoRMS) database (accessed December 2018). In the Hydrozoa class, only the species with a pelagic stage in their lifecycle were considered, following the species list of Reference [134].

Different taxonomic groups of jellyfish were studied for their associated microbiomes; but this review also reveals that the vast diversity of jellyfish as hosts remains to be explored (Figure 1). For instance, to our knowledge, not a single member of the Coronatae order was examined for its microbiome, while within Rhizostomeae, an entire suborder of Daktyliophorae remains unexplored and within Semaeostomeae, some members of the Drymonematidae family were never investigated for their microbiome. The WoRMS recognizes 69 genera of Scyphozoa comprising 191 species that populate different habitats, from tropical to polar regions, thus potentially harboring different microbes with unexplored biotechnological potential (Figure 1). Also, the Hydrozoa orders Actinulidae, Limnomedusae, Narcomedusae, and Trachymeduase remain to be investigated, and within Ctenophora several orders of the Tentaculata class remain to be explored.

Based on existing data, we tried to depict some basic characteristics of the jellyfish microbiome. It seems that the jellyfish microbiome is distinct from the bacterial community of the ambient water, comprising bacteria known for their preference for a surface-attached lifestyle and in association with marine organisms. This implies a certain degree of specialization of the microbiome of jellyfish, which are potentially generalists or possibly generalist symbionts. To some extent, it appears that the microbiome is jellyfish species-specific. However, in some instances, there is evidence that the jellyfish microbiome also depends on the background microbial community in the ambient water, possibly for recruiting members. This would also suggest a horizontal transmission of the microbiome to the outer body parts of the jellyfish. Accordingly, the microbiome associated with the outer body parts seems to exhibit a higher degree of similarity to the bacterial community in the ambient water and is also more diverse and variable than the microbiome associated with the inner compartments. It seems that the relationship between the microbiome of the inner body compartments and the jellyfish could be symbiotic, and that in this case, the mechanism of transmission could be vertical. The microbiomes of different life stages of jellyfish seem to vary, indicating a significant restructuring of the microbiome from the benthic to the pelagic stage of the jellyfish life cycle.

Our review of different methodological approaches used to study the jellyfish microbiome and the difficulties in comparing the available datasets calls for the establishment of more standardized and holistic sampling and analytic approaches. Also, as currently all hypotheses on the role and function of the jellyfish microbiome are rather speculative, we believe that metatranscriptomic, metaproteomics, and metabolomics should be applied, coupled with state-of-the-art microscopy techniques, to study the microbiome and the mechanisms underlying the associations to provide insight into the potential roles the microbiome might play in the ecology of jellyfish. Furthermore, we propose that investigations should be scaled down to the molecular level, i.e., the level at which microbial-mediated processes take place. Finally, regarding the biotechnological potential of the jellyfish-associated microbiome, the features of specific bacteria found to be associated with jellyfish that have potential application in blue biotechnology are, in fact, known characteristics of these bacterial strains or their closest relatives isolated from other marine organisms or substrates. To the best of our knowledge, no comprehensive study of biotechnological potential of the jellyfish-associated bacteria has been conducted to date. With this review, we provide insight into the jellyfish-associated microbiome and highlight its biotechnological potential, hoping to draw the attention of the blue biotechnology sector to explore jellyfish as a potentially great source of biotechnologically interesting microbes and the compounds they produce. Altogether, this will allow to fully exploiting the biotechnological potential locked in the jellyfish microbiome association.

## Figures and Tables

**Table 1:** Overview of publications on jellyfish-associated microbiome in terms of species studied (and their taxonomy) and jellyfish life stage, body compartment, sampling location, and methodology applied to analyze the composition and/or structure of the associated microbiome. FISH, fluorescence in situ hybridization; DGGE, denaturing gradient gel electrophoresis; ARISA, automated ribosomal intergenic spacer analysis; ITS, internal transcribed spacer; NGS, next-generation sequencing; T-RFLP, terminal restriction fragment length polymorphism.

Jellyfish Taxonomy					Study Design				
Phylum/Subphylum	Class	Order	Family	Species	Life Stage	Body Part (Adult Medusae)	Sampling Location	Methodology to Study Associated Microbiota	Publication
Medusozoa	Scyphozoa	Semaeostomeae	Ulmaridae	*Aurelia aurita*	Adult medusae	Whole body	North Atlantic coastal waters	16S rRNA gene clone libraries	[36]
					Polyps, strobila, ephyra, juvenile adult medusae	Mucus, gastric cavity	Kiel Bight, Baltic Sea, Southern English Channel, North Sea	Confocal laser scanning microscopy FISH NGS 454 technology V1–V2 16S rRNA region	[35]
					Adult medusae	Oral arms umbrella gastric cavity	Northern Adriatic	Culturing, DGGE, 16S rRNA gene clone libraries	[37]
			Cyaneidae	*Cyanea capillata*	Adult medusae	Tentacles	Scottish waters (Orkney)	Culturing DGGE, sequencing bands	[38]
				*Cyanea lamarckii*	Adult medusae	Tentacles	Scottish waters (Orkney)	Culturing DGGE, sequencing bands	[38]
					Larvae polyps adult medusae	Tentacles, umbrella, mouth arm, gonads	German Bight	ARISA of ITS region	[39]
			Pelagiidae	*Pelagia noctiluca*	Adult medusae	Mouth	Ireland	Sequencing of specific bacterial 16S rRNA gene	[33]
				*Chrysaora plocamia*	Polyps podocyst excyst	Whole body	Northern Chile	NGS Illumina MiSeq platform 2 × 300 bp paired end, V1–V2 16S rRNA region	[40]
				*Chrysaora hysoscella*	Larvae polyps adult medusae	Tentacles, umbrella, mouth arm, gonads	German Bight	ARISA of ITS region	[39]
		Rhizostomeae	Mastigiidae	*Mastigias cf. papua*	Adult medusae	Dome, tentacles	Indonesian marine lakes	NGS 454 technology, V3–V4 region	[41]
			Cepheidae	*Cotylorhiza tuberculata*	Adult medusae	Gastric cavity	Alcudia Bay, Balearic Sea	Culturing, NGS—454 pyrosequencing	[42]
					Adult medusae	Gastric cavity	Alcudia Bay, Balearic Sea	NGS—Illumina MiSeq platform, 2 × 250 bp, paired end	[43]
	Cubozoa	Carybdeida	Tripedaliidae	*Tripedalia cf. cystophora*	Adult medusae	Whole body	Indonesian marine lakes	NGS—454 technology V3-V4 16S rRNA region	[41]
	Hydrozoa	Anthoathecata	Bougainvilliidae	*Nemopsis bachei*	Adult medusae	Whole body	North Atlantic coastal waters	16S rRNA gene clone libraries	[36]
			Tubulariidae	*Tubularia indivisa*	Adult medusae	Tentacles	Scottish waters (Orkney)	Culturing DGGE, sequencing bands	[38]
		Leptothecata	Phialellidae	*Phialella quadrata*	Adult medusae	Whole body	Shetland Isles	Nested PCR with specific bacterial primers	[32]
					Adult medusae	Whole body	Ireland	RT PCR with specific bacterial primers	[34]
		Siphonophorae	Diphyidae	*Muggiaea atlantica*	Adult medusae	Whole body	Ireland	RT PCR with specific bacterial primers	[34]
Ctenophora	Tentaculata	Lobata	Bolinopsidae	*Mnemiopsis leidyi*	Adult specimen	Whole body	Tampa Bay, Florida, USA	16S rRNA gene clone libraries, T-RFLP	[44]
					Adult specimen	Whole body guts	Gullmar fjord, west coast of Sweden	NGS—454 pyrosequencing	[23]
					Adult specimen	Whole body	Helgoland roads, German Bight	ARISA of ITS region	[45]
				*Bolinopsis infundibulum*	Adult specimen	Whole body	Helgoland roads, German Bight	ARISA of ITS region	[45]
		Cydippida	Pleurobrachiidae	*Pleurobrachia pileus*	Adult specimen	Whole body	Helgoland roads, German Bight	ARISA of ITS region	[45]
	Nuda	Beroida	Beroidae	*Beroe ovata*	Adult specimen	Whole body	Helgoland roads, German Bight	ARISA of ITS region	[45]
					Adult specimen	Whole body	Tampa Bay, Florida, USA	16S rRNA gene clone libraries, T-RFLP	[44]

**Table 2:** Overview of dominant bacteria found to be associated with jellyfish and their attributed features with biotechnological potential.

Bacteria Associated with Jellyfish		Features with Biotechnological Potential	Jellyfish
Class	Representative Families		
*Gammaproteobacteria*	*Vibrionaceae* *Pseudoalteromonadaceae* *Alteromonadaceae* *Oceanospirillaceae* *Shewanellaceae* *Crenotrichaceae* *Methylococcalaceae* *Endozoicimonadaceae* *Moraxellaceae* *Legionelaceae*	- readily culturable - extremophiles - quorum-sensing factors/signals - degradation of high-molecular-weight compounds - antagonistic behavior - symbionts and/or pathogens - possibly stimulate the settlement of spores of other organisms (*Vibrio*) - producers of *ToxR* gene that mediate virulence expression (*Photobacterium profundum*) - producers of highly bioactive compounds (extracellular enzymes, exopolysaccharides, compounds involved in antimicrobial antifouling, with algicidal activity and various pharmaceutically relevant activities, e.g., *Pseudoaltermonas*) - multifunctional polyphenol oxidases, involved in secondary metabolism, biodegradation processes, breakdown of dimethylsulfoniopropionate (e.g., *Marinomonas*) - producers of antibacterial compounds - tetrodotoxin producers (*Pseudoalteromonas tetraodonis*) - septicemic, necrotic hemolytic, and cytolytic activity - produce polyunsaturated fatty acids (*Shewanella*)	- widespread among all studied jellyfish taxa; outer jellyfish body parts
*Alphaproteobacteria*	*Rhodospirillaceae* *Rhodobacteriaceae* *Kiloniellaceae*	- degradation of aromatic hydrocarbons; production of biosurfactants; utilization of hydrocarbons, carbohydrates, organic acids, or amino acids; and degradation of polycyclic aromatic hydrocarbons (e.g., *Thallasospira*) - readily culturable - found in association with living organisms and/or organic matter particles - found in sediment and microbial mats - inhibitory compound producers inhibit fouling of surfaces of host/substrate by interfering with cues for settlement of invertebrate larvae or spores of algae (e.g., *Phaeobacter*) - active antibiotic producers	- widespread among all studied jellyfish taxa
*Flavobacteria*	*Flavobacteriaceae*	- readily culturable - production/expression of various extracellular hydrolytic enzymes - degradation of complex organic materials - some pathogen representatives - some psychrophilic representatives - found in sewage-polluted waters, human guts (e.g., indicators of water quality) - carbohydrate and protein catalysis (e.g., *T. maritimum*)	- *A. aurita*, *P. noctiluca* and *C. plocamia*, *C. tuberculata gastric cavity* - *M. Leidyi* and *B. ovata* - Hydrozoa
*Mollicutes*	*Spiroplasmataceae* *Mycoplasmataceae*	- parasitic and commensal life-style - pathogens of humans, mammals, reptiles, fish, plants, and other arthropods and have been reported in different invertebrates, also cnidarians, such as corals - anaerobic fermenter metabolism	- *A. aurita* - *C.* *tuberculata gastric cavity* - Mastiigiidae - Cubozoa - ctenophores
*Spirochaetes*		- gut microbiota of marine animals - pathogens	- Mastigiidae - Cubozoa
*Actinobacteria*		- repertoire of enzymes to break down polysaccharides, proteins, and fats - rich source of secondary metabolites, antimicrobial and anticancer drugs	- *A. aurita* - *M. leidyi*
*Firmicutes*		- pathogens - bind and oxidize metals; bioremediation - powerful chitinolytic activity - source of toxins	- Cubozoa - ctenophores
*Chlamydiae*	*Simkania*-like bacteria	- pathogenic capability	- *C. tuberculata*’s gastric cavity
*Nitrospirae* and *Nitrospinae*		- potentially involved in regulating metamorphosis in marine invertebrates - potentially involved in regulating swimming of jellyfish - potential to facilitate discharge of nematocytes in *Aiptasia diaphana* - formation of bullet-shaped magnetite magnetosomes	- *C. plocamica*
*Betaproteobacteria*	*Burkholderia* *Achromobacter* *Cupriavidus*	- associated with polycyclic aromatic hydrocarbon degradation - associated with plastic degradation - associated with xenobiotic degradation - bioremediation and biopesticidal properties, - ability to synthesize wide range of antimicrobial compounds	- *A. aurita* - *C. plocamica* - *M. leiydi*
*Cyanobacteria*		- can survive extreme temperatures and salinities - production of secondary metabolites including exopolysaccharides, vitamins, toxins, enzymes, and pharmaceuticals - used in aquaculture, wastewater treatment, food, fertilizers - source of biologically active compounds with antiviral, antibacterial, antifungal, and anticancer activity - potential biofuel producers - removal of heavy metals from water, degrading oil components	- *C. tuberculata*’s gastric cavity - *A. aurita* - *M.leidyi*
